# Somatostatin Interneurons Recruit Pre- and Postsynaptic GABA_B_ Receptors in the Adult Mouse Dentate Gyrus

**DOI:** 10.1523/ENEURO.0115-24.2024

**Published:** 2024-08-14

**Authors:** Thomas C. Watson, Sam A. Booker

**Affiliations:** ^1^Simons Initiative for the Developing Brain, University of Edinburgh, Edinburgh EH8 9XD, United Kingdom; ^2^Centre for Discovery Brain Sciences, University of Edinburgh, Edinburgh EH8 9XD, United Kingdom

**Keywords:** astrocytes, dentate gyrus, GABA, GABA_B_, inhibition, somatostatin interneuron

## Abstract

The integration of spatial information in the mammalian dentate gyrus (DG) is critical to navigation. Indeed, DG granule cells (DGCs) rely upon finely balanced inhibitory neurotransmission in order to respond appropriately to specific spatial inputs. This inhibition arises from a heterogeneous population of local GABAergic interneurons (INs) that activate both fast, ionotropic GABA_A_ receptors (GABA_A_R) and slow, metabotropic GABA_B_ receptors (GABA_B_R), respectively. GABA_B_Rs in turn inhibit pre- and postsynaptic neuronal compartments via temporally long-lasting G-protein-dependent mechanisms. The relative contribution of each IN subtype to network level GABA_B_R signal setting remains unknown. However, within the DG, the somatostatin (SSt) expressing IN subtype is considered crucial in coordinating appropriate feedback inhibition on to DGCs. Therefore, we virally delivered channelrhodopsin 2 to the DG in order to obtain control of this specific SSt IN subpopulation in male and female adult mice. Using a combination of optogenetic activation and pharmacology, we show that SSt INs strongly recruit postsynaptic GABA_B_Rs to drive greater inhibition in DGCs than GABA_A_Rs at physiological membrane potentials. Furthermore, we show that in the adult mouse DG, postsynaptic GABA_B_R signaling is predominantly regulated by neuronal GABA uptake and less so by astrocytic mechanisms. Finally, we confirm that activation of SSt INs can also recruit presynaptic GABA_B_Rs, as has been shown in neocortical circuits. Together, these data reveal that GABA_B_R signaling allows SSt INs to control DG activity and may constitute a key mechanism for gating spatial information flow within hippocampal circuits.

## Significance Statement

GABAergic interneurons provide powerful inhibition to cortical circuits, by directly regulating the activity of other neurons through metabotropic GABA_B_ receptors. While much is known about the basic properties of GABA_B_ receptor signaling, current knowledge of the relative contribution made by specific interneuron subpopulations to this inhibitory neurotransmission mechanism is less well understood. Our results help address this knowledge gap by showing that the somatostatin-expressing interneuron subpopulation provide powerful GABA_B_ receptor-mediated feedback inhibition in the mouse dentate gyrus. Furthermore, GABA_B_ receptor activation in dentate gyrus was found to be tightly regulated by neuronal GABA uptake, and not astrocytes, thus providing self-regulated feedback inhibition. Together, these data provide novel insights into cell type-specific GABA_B_ receptor-mediated control of dentate gyrus circuitry.

## Introduction

Spatial information processing in the mammalian dentate gyrus (DG) requires dynamic and inhibitory GABAergic neurotransmission from local interneurons (INs) to control the timing of synaptic information arising from entorhinal cortex input (Pernía-Andrade and Jonas, 2014) in addition to controlling the excitability of dentate granule cells (DGCs; Hainmueller and Bartos, 2020). In the DG, INs release GABA from axons that are aligned in a layer-specific manner (Degro et al., 2022), which in turn activates GABA receptors found on both DGC and IN pre- and postsynaptic membranes (Kulik et al., 2018). In DGCs, the mechanisms subserving GABA_A_ receptor (GABA_A_R)-mediated inhibitory influence on circuit function are well described (Bartos et al., 2001; Vida et al., 2006). While temporally slow, metabotropic GABA_B_ receptors (GABA_B_Rs) have been described in DGCs (Mott et al., 1993, 1999; Degro et al., 2015), their activation mediated by different IN subtypes is less well understood.

GABA_B_Rs activate Kir3 channels (Bettler et al., 2004) and inhibit Ca^2+^ channels in dendrites and axons (Vigot et al., 2006). This modulation pattern shows cell- and region-specific differences, including in the DG (Mott and Lewis, 1991; Mott et al., 1993, 1999; Piguet, 1993; Brucato et al., 1995; Lüscher et al., 1997; Booker et al., 2013, 2017a, 2018, 2020; Foster et al., 2013; Degro et al., 2015). GABA_B_Rs are typically located outside of the synaptic cleft (Kulik et al., 2003). They are activated by GABA spillover, which allows for coordination of circuit activity over hundreds of milliseconds (Isaacson et al., 1993; Scanziani, 2000; Gonzalez-Burgos et al., 2009). This heterosynaptic nature suggests that INs can potentially activate neuronal GABA_B_Rs independently of direct synaptic connections (Scanziani, 2000; Degro et al., 2015) to regulate wider circuit function over longer timescales.

Indeed, some INs (e.g., neurogliaform cells—NGFCs) can recruit GABA_B_R in a unitary manner (Thomson and Destexhe, 1999; Tamás et al., 2003; Price et al., 2005, 2008; Oláh et al., 2009). However, neuronal circuits rarely function in isolation and rather require coordinated activation of many neurons (Klausberger et al., 2003). INs that express somatostatin (SSt) are readily recruited by active circuits and provide powerful feedback inhibition over multiple timescales in both hippocampus and neocortex (Pangalos et al., 2013; Urban-Ciecko et al., 2015; Booker et al., 2020; Honoré et al., 2021). In the DG, SSt INs are located in the hilus (Savanthrapadian et al., 2014; Booker and Vida, 2018) where they release GABA onto the distal dendrites of granule cells (GCs), aligned to entorhinal cortex (EC) inputs (Booker and Vida, 2018; Degro et al., 2022) to dynamically control DG circuit activity (Hainmueller et al., 2024). Indeed, the axonal output of SSt INs very closely aligns with the highest expression of GABA_B_Rs and effector Kir3 channels on DGC dendrites (Degro et al., 2015). This indicates that SSt INs are ideally positioned to exert powerful GABA_B_R inhibition on DGCs. Evidence does exist for SSt INs to recruit pre- and postsynaptic GABA_B_Rs (Urban-Ciecko et al., 2015; Gonzalez et al., 2018; Kanigowski et al., 2023). However, the mechanisms by which SSt INs recruit spillover of GABA to activate GABA_B_Rs, whether sex differences exist in signaling, or how presynaptic receptors modulate DG function remain unexplored.

In this study we hypothesize that SSt INs can activate pre- and postsynaptic GABA_B_Rs in the mouse DG and that this activation is tightly regulated by GABA uptake mechanisms. To test this, we virally transfected channelrhodopsin 2 into the DG of mice expressing Cre-recombinase under the SSt promotor channel. Using ex vivo whole-cell electrophysiology, we show that SSt INs recruit a significant postsynaptic GABA_B_R-mediated current in DGCs. DG GABA_B_R signaling displayed some sex dependence and was predominantly regulated by neuronal GABA uptake. Finally, we confirm that SSt INs can recruit presynaptic GABA_B_Rs to regulate neurotransmitter release, as previously described in neocortex.

## Materials and Methods

### Animals

Electrophysiological experiments were performed in acute slices prepared from adult (3–7-month-old) transgenic mice of both sexes expressing Cre-recombinase under the SSt promoter (SSt^tm2.1(cre)Zjh/J^; Jackson Laboratory). All mice were housed in groups of 2–4 prior to surgery, maintained on a 12 h light/dark cycle. Under anesthesia (2% isoflurane), either unilateral or bilateral injections of AAV containing channelrhodopsin 2 (ChR2), and YFP coding regions between inverted incompatible tandem loxP sites (AAV2-FLEX-EF1a-DIO-hChR2(H134)-EYFP, ≥7 × 10^12^ vg/ml, Addgene) were made in to the DG (coordinates relative to the bregma: −2 mm posterior, 1.5 mm lateral and 1.5 mm ventral from brain surface; 500 nl volume). Slice recordings were performed ∼2 weeks following viral injection (12–17 d, median 14 d) at zeitgeber time 2–3. All animal procedures were performed in accordance with United Kingdom Home Office (ASPA 1986) and the University of Edinburgh animal care committee regulations.

### Brain slice preparation and recording

Mice were sedated with isoflurane, anesthetized with sodium pentobarbital (20 mg/kg), and then transcardially perfused with ice-cold, carbogenated (95% O_2_/5% CO_2_) sucrose artificial cerebrospinal fluid (sucrose-ACSF, in mM: 87 NaCl, 2.5 KCl, 25 NaHCO_3_, 1.25 NaH_2_PO_4_, 25 glucose, 75 sucrose, 7 MgCl_2_, 0.5 CaCl_2_, 1 Na-pyruvate, 1 Na-ascorbate), and then the brain was rapidly dissected. Coronal hippocampal slices (300 μm) were cut on a vibratome (VT1200s, Leica) and stored in sucrose-ACSF at 35°C for 30 min and then at RT until recording, as previously described (Oliveira et al., 2021).

For recording, slices were placed in a submerged recording chamber, perfused with carbogenated ACSF (in mM: 125 NaCl, 2.5 KCl, 25 NaHCO_3_, 1.25 NaH_2_PO_4_, 25 glucose, 1 MgCl_2_, 2 CaCl_2_, 1 Na-pyruvate, 1 Na-ascorbate) at 6–8 ml/min maintained at near physiological temperatures (32 ± 1°C). Slices were visualized with IR-DIC with an upright microscope (SliceScope, Scientifica) with a 40× water-immersion objective lens (NA 0.8). Whole-cell patch-clamp recordings were accomplished using a MultiClamp 700B amplifier (Molecular Devices) with recording pipettes pulled from borosilicate glass capillaries (1.5 mm outer/0.86 mm inner diameter, Harvard Apparatus) on a horizontal electrode puller (P-97, Sutter Instruments). Pipettes were filled with intracellular solution that gave pipette resistances of 3–7 MΩ. In all recordings series resistance (*R_s_*) was monitored, which was not compensated in voltage clamp. In current-clamp mode, the bridge was fully balanced. All signals were filtered online at either 2 kHz (voltage-clamp) or 5 kHz (current-clamp) using a two-pole Bessel filter and digitized at 20 kHz (Digidata 1550B, Axon Instruments) using pClamp 11 (Axon Instruments). Data were analyzed off-line using the open source Stimfit software package (Guzman et al., 2014). Neurons were rejected from further analysis when *V_M_* > −50 mV, if APs failed to overshoot 0 mV, initial *R_s_* exceeded 30 MΩ, or *R_s_* changed by >20% in the course of the recording.

DGCs were selected for recording on the basis of having small, round somata located in the GCL. For GABA_B_R IPSC recordings, electrodes were filled with an K-gluconate intracellular solution (in mM: 142 K-gluconate, 4 KCl, 2 MgCl_2_, 0.2 EGTA, 10 HEPES, 2 Na_2_-ATP, 0.3 Na_2_-GTP, 10 Na_2_-phosphocreatine, 0.1% biocytin, pH 7.35, 290–310 mOsm). DGCs were first electrophysiologically characterized based on their response to a family of hyper- to depolarizing current injections (500 ms duration; −500 to 500 pA in 100 pA steps) from resting *V_M_*. Combined optogenetic and electrical stimulation experiments were performed in ACSF containing NBQX and AP-5 (10 and 50 µM, respectively) in voltage clamp at −60 mV. Optogenetic stimulation was performed using 473 nm LED light (CooLED) delivered to the whole field of view centered on the outer molecular layer (oML). Brief (5 ms) pulses of light of varying intensity (0–50% LED power) were used to deliver to the slice either singly or in trains of five stimuli at 50 Hz. For electrical stimulation, bipolar twisted Ni:Chrome electrodes were placed in the oML (∼500 μm distal to the recorded cell) and IPSCs elicited by five stimuli at 200 Hz (200 μs, 50 V). Following initial characterization of compound IPSCs (5–10 sweeps), 10 μM gabazine (SR-95,531) was bath applied to block GABA_A_R-mediated currents, and then a further 15 IPSCs were collected (at 20 s intervals). Following this baseline recording, 20 μM tiagabine, 100 μM SNAP-5,114, or 5 μM CGP-55,845 were applied to the bath. In a subset of recordings, slices from the contralateral hemisphere, which lacked strong ChR2-YFP expression, were recorded for electrical IPSCs only. In these recordings, following IPSC characterization 10 μM *R*-baclofen (a selective GABA_B_R agonist) was bath applied for 5 min, followed by 5 μM CGP-55,845 for 5 min. In a subset of cells, we assessed the reversal potential of GABA_B_R IPSCs following baseline optogenetic and electrical IPSC recordings. For this, the DGCs were voltage clamped at a variety of membrane potentials (−40 to −100 mV) and three IPSCs elicited at each level. Holding potentials were adjusted for the liquid junction potential, measured at 12 mV. For all IPSCs, the amplitude was measured over a 10 ms average window. IPSC kinetics was measured from average IPSCs. A total of 20–80% IPSC rise time was measured from the prestimulus baseline. Decay time constant was estimated from the fit of a monoexponential curve fitted to the average IPSC.

For presynaptic experiments, DGCs were recorded at −70 mV voltage-clamp using a Cs-gluconate–based internal solution (in mM: 140 Cs-gluconate, 3 CsCl, 0.2 EGTA, 10 HEPES, 2 Na_2_-ATP, 2 Mg-ATP, 0.3 Na_2_-GTP, 10 Na_2_-phosphocreatine, 5 QX-314.Cl, 0.1% biocytin, pH 7.35, 290–310 mOsm) using ACSF containing 10 μM gabazine. Excitatory postsynaptic currents (EPSCs) were elicited by placing a bipolar, twisted Ni:Chrome wire placed in the oML, and trains of stimuli delivered at either 20 or 50 Hz (5 or 13 stimuli, respectively). Stimulus strength (mean, 22.7 V; range, 5–50 V) was chosen to generate a monosynaptic EPSC (mean, −127 pA; range, −27 to −356 pA). For co-activation of SSt INs, optogenetic stimulation 473 nm LED light was delivered at 20% maximum power. Five-millisecond optogenetic stimulation was delivered concomitant with each electrical stimulus. Following wash-in of the internal solution, 10 EPSCs were collected for each frequency stimulus train (10 s intervals) with or without optogenetic activation, then 5 μM CGP-55,845 was bath applied for 5 min, and then trains of stimuli were delivered again. In a subset of recordings, gabazine was omitted from the ACSF and neurons recorded at 0 mV, to measure the monosynaptic IPSC resulting from ChR2 activation, in the absence of electrical stimulation. In these recordings, a minimum of 30 sweeps were collected (10 s intervals), and then 5 μM CGP-55,845 was applied to the bath. For experiments blocking G_i/o_ activation, slices were incubated in recording ACSF containing 500 µg/ml pertussis toxin for 2–3 h prior to transfer to the recording chamber. For analysis, the amplitude of each EPSC was measured as the peak response (2.5 ms average window) relative to the prestimulus baseline. Short-term plasticity (STP) is plotted as the amplitude of EPSCs in the stimulus train normalized to the first EPSC.

### Visualization, imaging, and reconstruction of the recorded neurons

Following recording, neurons were sealed with outside-out patches and fixed in 4% paraformaldehyde diluted in 0.1 M PB overnight (O/N) at 4°C. Immunohistochemistry was performed as previously described (Booker et al., 2014). Briefly, slices were washed with phosphate-buffered saline (PBS; 0.025 M PB and 0.9% NaCl) and then blocked with 10% normal goat serum (NGS) with 0.5% Triton X-100 and 0.05% NaN_3_ diluted in PBS for 1 h at RT. Slices were then incubated for 72 h in a PBS solution containing 5% NGS, 0.3% Triton X-100, and 0.05% NaN_3_ and primary antibodies against SSt (rabbit polyclonal, 1:500, Peninsula Laboratories), at 4°C. Slices were then washed in PBS for an hour and then incubated with fluorescently conjugated secondary antibodies raised against rabbit (goat anti-rabbit Alexa Fluor 568; 1:500, Thermo Fisher Scientific) as well as fluorescently conjugated streptavidin (Alexa Fluor 633; 1:500, Thermo Fisher Scientific) to visualize recorded neurons, in a PBS solution containing 3% NGS, 0.1% Triton X-100%, and 0.05% NaN_3_ overnight at 4°C. Slices were rinsed in PBS and then 0.1 M phosphate buffer containing 1 μg/ml DAPI and mounted on glass slides (Fluoromount-G, SouthernBiotech). Filled neurons were imaged with a laser scanning confocal microscope (SP8, Leica) using a 20× objective lens (NA 0.75). For quantification of SSt/ChR2-YFP overexpression, *z*-axis stacks of images (2,048^2^ pixels, 1 μm steps) cover the extent of the slice (1 slice/injected hemisphere/mouse). Selected, example cells were imaged for confirmation of cell type. All image analysis was performed using FIJI (ImageJ, http://fiji.sc).

### Statistical analysis

**Figure 1.**
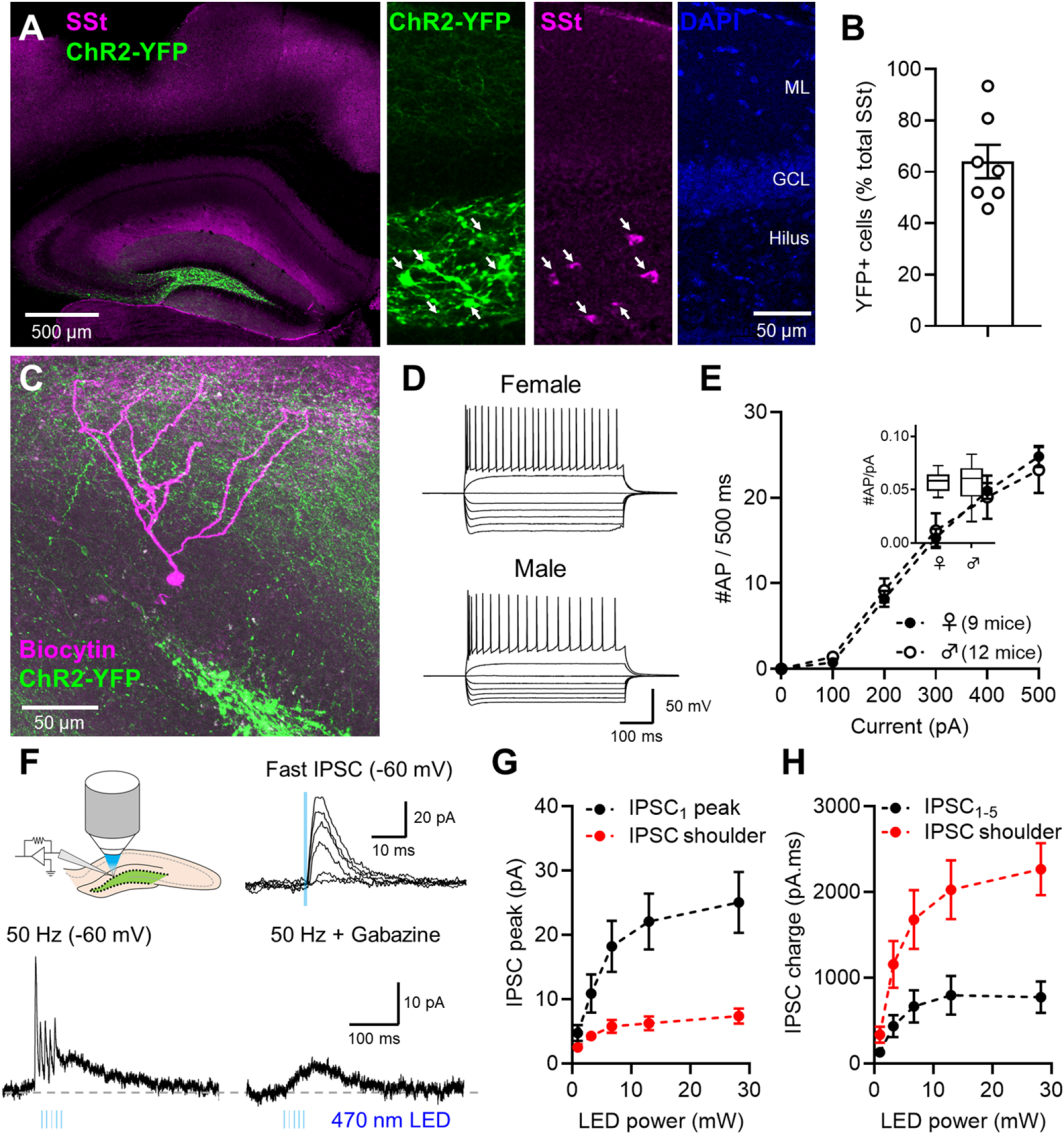
Expression of ChR2 in SSt-Cre mice leads to robust inhibitory input to DGCs, which is composed of fast and slow inhibition. ***A***, Overview of low-magnification confocal image showing expression of ChR2-YFP (green) with respect to SSt immunolabeling (magenta). Right, Higher magnification images showing ChR2-YFP (green) expression in somata (arrows) and dendrites of the hilus, with sparse axons in the ML, which largely colocalized with SSt immunolabeling (magenta), compared with DGC layering (blue). ***B***, Quantification of the percentage of all SSt immunolabeled neurons that were positive for ChR2-YFP in the hilus of the DG from seven mice. ***C***, Flattened confocal stack of a recorded and biocytin-filled DGC (magenta) with respect to ChR2-YFP labeling (green). ***D***, Example voltage responses to hyper- and depolarizing (−500 to +500 pA, 100 pA steps, 500 ms duration) of DGCs from adult male and female mice. ***E***, Current-frequency plot of action potential (AP) discharge from male (open circles, 9 mice) and female (black circles, 9 mice) DGCs. Inset, Quantification of neuronal gain (#AP/pA) for female (*n* = 9 mice) and male (*n* = 12 mice) mice (*U*_(21) _= 51; *p* = 0.862; Mann–Whitney test). ***F***, Schematic overview of experimental setup indicating optogenetic stimulation (left), with example traces of fast IPSCs in response to increasing LED power (top) and following high-frequency (50 Hz, 13 mW) optical stimulation in control ACSF (left) or the presence of 10 µM gabazine (right). ***G***, Amplitude comparison of fast IPSCs (black) and the slow IPSC shoulder (red) in recorded DGCs in response to increasing LED power. ***H***, Comparison of charge transfer for fast IPSCs (black) and slow IPSC shoulder (red) in response to increasing LED power. All data is shown as mean ± SEM.

All data is shown from cell average replicates as 1–2 cells per condition being collected per biological replicate (individual mouse); thus it was not possible to obtain an estimate of intra-animal variability. All graphs display data generated from individual cells, except for Fig. 1*B*,*E*, which display animal average data. Mice of either sex were used throughout. As all mice expressed Cre-recombinase under the SSt-cre promotor, and ChR2-YFP expression was assessed prior to recording, it was not possible to be blind to virus expression. Throughout, all data is shown as the mean ± SEM (except for Table 1). Statistical analysis was performed with GraphPad Prism (GraphPad Software). Analysis of unpaired data was performed with Student's *t* test or Mann–Whitney *U* tests depending on whether data were normally distributed (D’Agostino–Pearson test). Group data were compared with two-way ANOVA tests, combined with Sidak post-tests. Statistical significance was assumed if *p* < 0.05.

## Results

### DG SSt INs directly recruit multiple GABA receptors

A key primary aim of this study was to determine whether SSt INs are capable of activating postsynaptic GABA_B_Rs. To address this, we expressed channelrhodopsin 2 (ChR2-YFP) in SSt INs in the dorsal DG and then prepared coronal brain slices ∼14 d after AAV injection (range, 12–19 d). Viral expression was confirmed by the presence of YFP-positive somata restricted to the hilus and with dense axon collaterals in molecular layers (MLs; Fig. 1*A*).

When compared with immunohistochemical labeling, we observed that 64.0 ± 6.1% of SSt neurons expressed ChR2-YFP (*n* = 7 mice; Fig. 1*B*). This labeling was highly specific, with few (3.2 ± 1.2%) neurons expressing ChR2-YFP, but without detectable SSt. To directly measure GABA_B_R currents, we performed whole-cell patch-clamp recordings from identified DGCs from male (*n* = 9 mice) and female (*n* = 12 mice; Fig. 1*C*). DGCs did not display sex-specific differences in action potential discharge (Fig. 1*D*,*E*) or most other basal electrophysiological properties, with the exception of action potential threshold (Table 1).

**Table 1.** Summary of DGC electrophysiological properties recorded from male and female mice

Physiological parameter	Female *n* = 30 cells, *N* = 9 mice	Male *n* = 22 cells, *N* = 12 mice	*p* value
Resting membrane potential (mV)	−79.6 ± 3.0	−78.4 ± 5.4	0.501
Input resistance (MΩ)	140.4 ± 18.8	147.3 ± 45.3	0.641
Membrane time constant (ms)	8.6 ± 1.5	8.5 ± 3.7	0.977
Membrane capacitance (pF)	63.3 ± 16.0	63.4 ± 32.4	0.995
Rheobase (pA)	187 ± 23	180 ± 58	0.673
AP voltage threshold (mV)	−39.5 ± 3.2	−34.5 ± 4.9	**0.012**
AP amplitude (mV)	128.2 ± 4.0	126.5 ± 10.6	0.622
AP ½ height duration (ms)	0.70 ± 0.06	0.70 ± 0.11	0.771
AP 20–80% rise time (ms)	0.12 ± 0.02	0.12 ± 0.03	0.821
AP maximum rise (mV·ms^−1^)	546 ± 82	522 ± 91	0.536
AP maximum decay (mV·ms^−1^)	117 ± 9	117 ± 15	0.961
*F*–*I* slope (AP·pA^−1^)	0.057 ± 0.009	0.057 ± 0.018	0.970

All data are shown as mean ± SD from cell averages—reflecting cellular variance. Statistics shown as *p* values from Student's two-tailed, unpaired *t* test from animal average data, to avoid type 1 statistical errors. Statistically significant values are shown in bold.

SSt INs possess axons primarily ramifying in the oML (Hosp et al., 2014; Yuan et al., 2017; Booker and Vida, 2018; Degro et al., 2022) which coaligns with high-density GABA_B_R and Kir3 channel expression (Degro et al., 2015). In whole-cell patch-clamp recordings performed in DGCs from −60 mV, we optogenetically stimulated slices over the oML to recruit dendritic GABAergic currents originating from SSt IN activation. Single stimuli (5 ms duration) resulted in 13.6 ± 2.2 pA currents, which had a fast rise, slow decay—consistent with GABA_A_R activation at −60 mV (*E_R_*[Cl^−^] ≈ −72 mV; Fig. 1*F*). Trains of five stimuli (50 Hz) resulted in both fast and slow IPSCs, the latter of which lasted several hundred milliseconds and remained following bath application of the GABA_A_R antagonist gabazine (10 μM). The peak amplitude of fast and slow IPSCs largely tracked each other with increasing stimulation (Fig. 1*G*), albeit with smaller shoulder currents [*F*_(1,80) _= 41.68; *p *< 0.0001; two-way ANOVA (current type); *n* = 9 cells from 3 mice]. However, when charge of IPSCs was measured, it was apparent that GABA_B_R-mediated currents from SSt IN optogenetic stimulation were significantly larger (as measured from the soma at −60 mV) than for GABA_A_Rs [*F*_(1,80) _= 39.85; *p *< 0.0001; two-way ANOVA (current type); Fig. 1*H*].

### GABA release from SSt INs recruits GABA_B_R-Kir3 currents and which is regulated by neuronal uptake mechanisms

**Figure 2.**
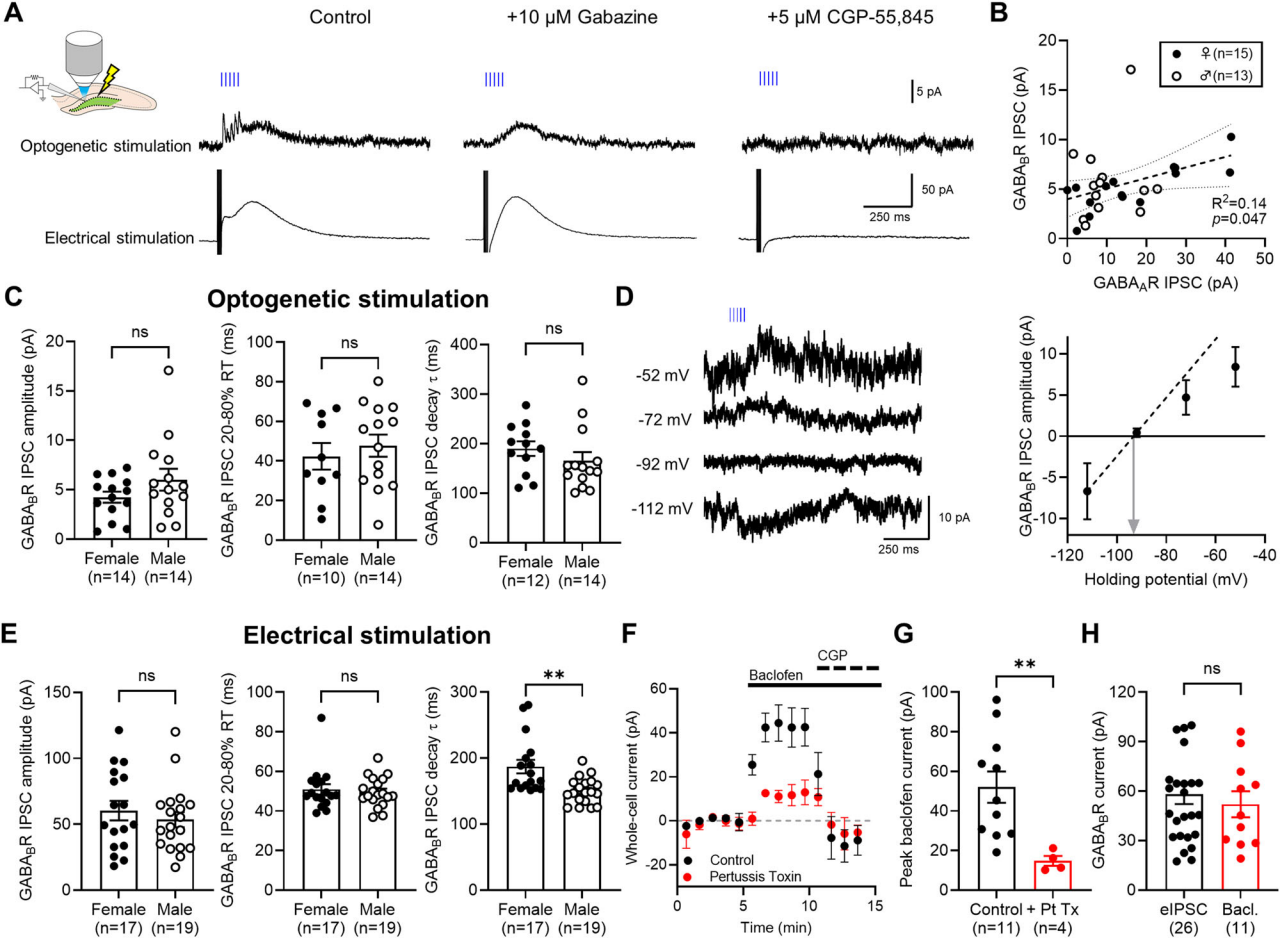
Postsynaptic GABA_B_R-mediated currents originating from SSt INs in adult mouse dentate gyrus. ***A***, Schematic overview of experimental setup indicating combined optogenetic and electrical stimulation (left). Example traces from a recorded DGC in which both SSt-mediated optogenetic IPSCs (blue lines, top) and electrically stimulated IPSCs (5× 200 Hz, 50 V, bottom) are shown for each pharmacological epoch tested. ***B***, Comparison of fast GABA_A_R-mediated IPSC amplitude, with GABA_B_R IPSCs from both sexes. Linear regression is shown for the whole population (dashed line, 95% confidence interval), with *R*^2^ stated on the graph. ***C***, Quantification of the amplitude, 20–80% rise time (RT), and decay time constant (*τ*) of optogenetic-evoked GABA_B_R-mediated IPSCs from DGCs recorded from adult female (closed circles; *n* = 14 cells from 7 mice) and male (black circles; *n* = 14 cells from 8 mice) animals. We found no difference in GABA_B_R IPSC amplitude (*U*_(28) _= 74; *p* = 0.285; Mann–Whitney test) or 20–80% RT (*U*_(28) _= 61; *p* = 0.625; Mann–Whitney test) or decay *τ* (*U*_(28) _= 56; *p* = 0.160; Mann–Whitney test). ***D***, Optogenetic stimulated GABA_B_R-mediated IPSCs recorded at a range of holding potentials (indicated on left), adjusted for liquid junction potential. Summary plot of optogenetic stimulated GABA_B_-mediated IPSC amplitude for the range of holding potentials, with linear fit of inward and zero currents. Reversal potential is indicated (gray arrow). ***E***, Quantification of the average amplitude, 20–80% rise time (RT), and decay time constant (*τ*) of electrically evoked GABA_B_R-mediated IPSCs from DGCs recorded from adult female (closed circles; *n* = 17 cells from 8 mice) and male (black circles; *n* = 18 cells from 8 mice) animals, following stimulation of oML in the presence of CNQX (10 µM), AP-5 (50 μM), and gabazine (10 μM). We found no difference in GABA_B_R IPSC amplitude (*U*_(34) _= 132; *p* = 0.5034; Mann–Whitney test) or 20–80% RT (*U*_(34) _= 146; *p* = 0.8323; Mann–Whitney test), albeit, decay *τ* was longer in female mice (*U*_(34) _= 58; *p* = 0.0012; Mann–Whitney test). ***F***, Pharmacological activation of GABA_B_Rs following bath application of *R*-baclofen (10 μM), followed by subsequent CGP-55,845 (5 μM) wash-in. Data is shown for control recordings (black; from 11 cells from 10 mice) and from slices preincubated with 500 μg/ml pertussis toxin (red, 3 cells from 3 mice). ***G***, Pertussis toxin preapplication (*n* = 4 cells from 3 mice) significantly reduced *R*-baclofen–mediated currents (black; *n* = 11 cells from 10 mice; *U*_(15) _= 1; *p* = 0.0029; Mann–Whitney test). ***H***, Comparison of the peak electrical GABA_B_R IPSC (black, 26 cells from 16 mice) and baclofen mediated currents (11 cells from 10 mice; *U*_(37) _= 123; *p* = 0.523; Mann–Whitney test). All data is shown as mean ± SEM. Statistics shown: ^ns^*p* > 0.05, **p < 0.01, ***p < 0.001; from Mann–Whitney *U* tests.

To pharmacologically identify the slow IPSCs, we optogenetically stimulated slices (5 × 50 Hz) while recording interleaved electrically evoked IPSCs resulting from oML electrical stimulation (5 × 200 Hz stimuli, 50 V amplitude). Both optogenetic and electrical stimulated IPSCs displayed a fast and slow IPSC. Fast IPSCs were mediated by GABA_A_R due to their sensitivity to gabazine, leaving only slow outward currents which were blocked by the selective antagonist CGP-55,845 (CGP; 5 μM), confirming they were mediated by GABA_B_Rs (Fig. 2*A*). In some cells, GABA_B_R-mediated IPSCs were observed even in the absence of SSt IN-mediated GABA_A_R responses (2 out of 28 cells), indicating a heterosynaptic nature of GABA_B_R activation (Fig. 2*B*). GABA_B_R-mediated optogenetic IPSCs had amplitudes of 4.95 ± 0.59 pA, with slow 20–80% rise (45.4 ± 4.3 ms) and long decay time constants (173.2 ± 11.7 ms; *n* = 31 cells from 15 mice; Fig. 2*C*). We observed no difference in amplitude (*U*_(27) _= 74; *p *= 0.285; Mann–Whitney test) or rise time (*t*_(22) _= 0.616; *p *= 0.545; Student's two-tailed *t* test) between male (*n* = 14 cells from 8 mice) and female mice (*n* = 14 cells from 7 mice). GABA_B_R decay time constants were typically longer in female mice, compared with males, albeit not significantly so (*U*_(25) _= 56; *p *= 0.159; Mann–Whitney test). We confirmed that SSt IN-driven GABA_B_R-mediated activation led to Kir3 channel opening, as slow IPSCs had a reversal potential of −93.2 mV and displayed clear inward rectification depolarized potentials (*n* = 6 cells from 5 mice; Fig. 2*D*). Electrical stimulation of oML produced similar GABA_B_R-mediated IPSCs, albeit they were substantially larger (Fig. 2*E*). Notably, GABA_B_R-mediated IPSCs evoked by electrical stimulation in female mice (*n* = 17 cells from 8 mice) had an average decay time constant of 187.2 ± 42.3 ms, which was significantly slower than that of male mice (150.1 ± 4.8 ms; *n* = 18 cells from 8 mice; *U*_(34) _= 58; *p *= 0.0012; Mann–Whitney test). Whole-cell GABA_B_R-mediated responses in DGCs were activated by the agonist baclofen (10 μM), blocked by CGP, and were sensitive to pertussis toxin; which blocks G_i/o_ activation (Fig. 2*F*). Indeed, pertussis toxin pretreatment reduced the average peak baclofen current by 72% (*U*_(14) _= 1; *p *= 0.0029; Mann–Whitney test; Fig. 2*G*). Comparison of electrically evoked GABA_B_R IPSCs and peak baclofen-mediated whole-cell currents revealed that direct stimulation of endogenous GABA release in oML (recruiting all dendritic targeting INs) activated all DGC receptors (*U*_(36) _= 123; *p *= 0.523; Mann–Whitney test; Fig. 2*H*). Together, these data confirm that SSt INs recruit a substantial proportion of native GABA_B_Rs, which are largely similar between sexes—but with subtle changes in decay kinetics.

**Figure 3.**
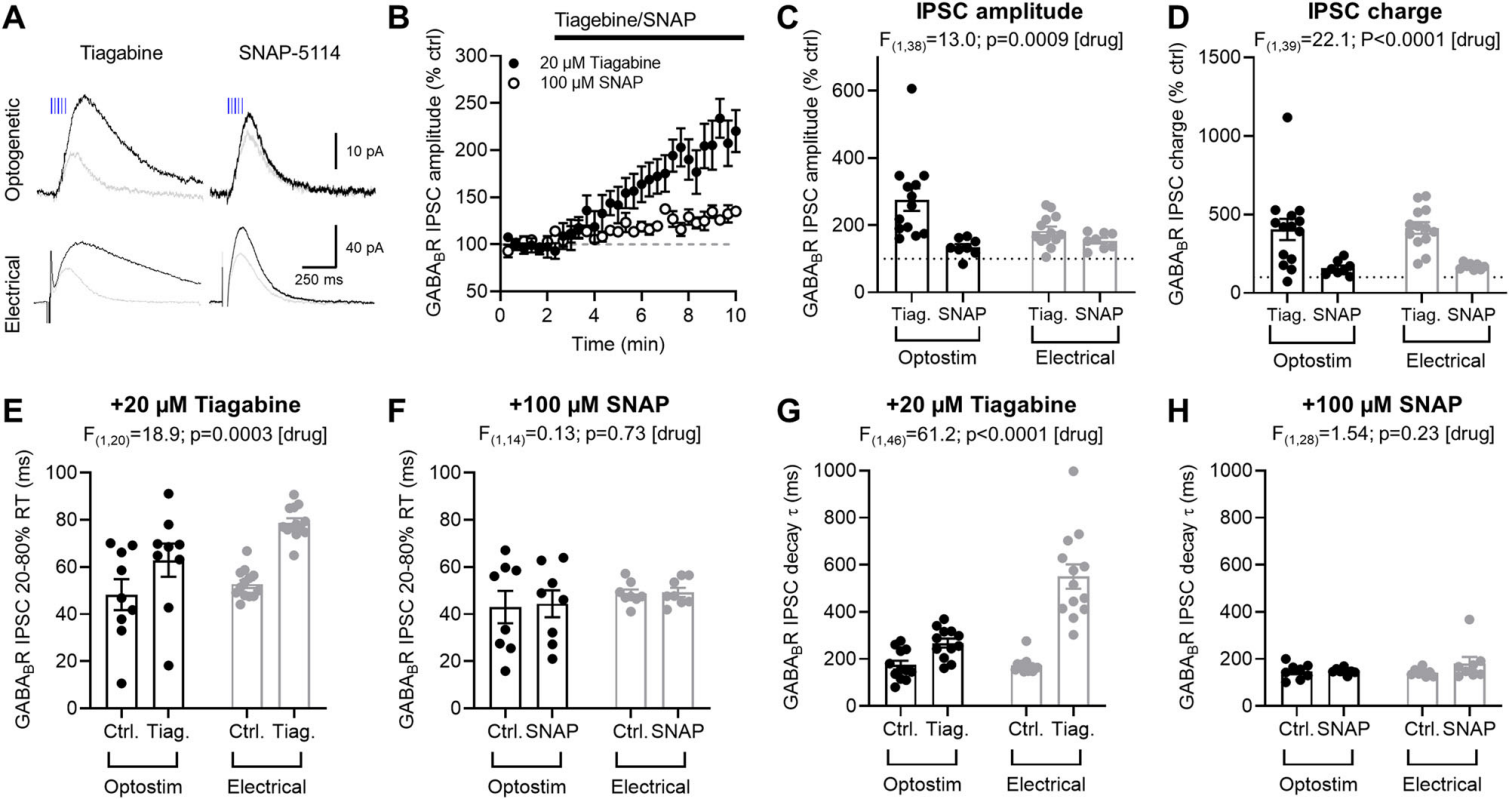
Neuronal, not astrocytic, GABA uptake controls GABA_B_R signaling in DGCs from adult mice, independent of presynaptic source. ***A***, Example traces of optogenetic and electrically stimulated GABA_B_R-mediated IPSCs before (gray) or after (black) bath application of 20 μM tiagabine (left) or 100 μM SNAP-5,114 (right). ***B***, Time course of optogenetic stimulated GABA_B_R-mediated IPSC amplitudes before and after (black bar) bath application of either tiagabine (Tiag., filled circles) or SNAP-5,114 (SNAP, open circles), as compared with the baseline period (gray dashed line). ***C***, Quantification of GABA_B_R IPSC amplitude modulation by GAT1 and GAT3 inhibitors for optogenetic stimulation (Optostim; black) or electrical (gray) stimulation. ***D***, Quantification of GABA_B_R IPSC charge transfer modulation by GAT1 and GAT3 inhibitors. ***E***, Bath application of the GAT1 inhibitor tiagabine (20 μM; *n* = 14 cells from 11 mice) prolonged the 20–80% rise time (RT) of SSt IN and electrically evoked GABA_B_R-mediated IPSCs. ***F***, Bath application of the GAT3 inhibitor SNAP-5,114 (100 μM; *n* = 8 cells from 6 mice) had no effect on GABA_B_R-mediated IPSC rise time, for either stimulation method. ***G***, Tiagabine strongly increased GABA_B_R-mediated IPSC decay time constants [*τ*; *F*_(1,23) _= 24.30; *p *< 0.0001; 2-way repeated-measures ANOVA (interaction)], which produced longer-lasting IPSCs following electrical stimulation, compared with SSt INs alone (*t*_(46) _= 6.619; *p *< 0.0001; Holm–Sidak test). ***H***, SNAP-5,114 had no effect on decay time constants of GABA_B_R-mediated IPSCs, irrespective of stimulation method. All data is shown as mean ± SEM, with data from individual cells overlaid. Statistics shown as main effects of two-way repeated-measures ANOVA above graphs.

GABA_B_R activation is driven by heterosynaptic spillover of GABA (Isaacson et al., 1993; Scanziani, 2000), which may be mediated by neuronal (Gonzalez-Burgos et al., 2009; Gonzalez et al., 2018) or astrocytic uptake mechanisms (Matos et al., 2018; Shen et al., 2022). To determine if spillover of GABA contributes to GABA_B_R signaling in DGCs, we blocked transporters located on either neurons (GAT-1) or astrocytes (GAT-3), with tiagabine or SNAP-5,114, respectively (Fig. 3*A*,*B*). Tiagabine bath application substantially increased the amplitude of optogenetic SSt IN mediated (247 ± 32% of control) and electrically-evoked GABA_B_R-mediated IPSCs (183 ± 13% of control; Fig. 3*C*). This was mirrored in a substantial increase in GABA_B_R-mediated charge transfer of SSt IN (405 ± 71% of control; *n* = 14 cells from 11 mice) and electrically evoked responses (414 ± 38% of control, Fig. 3*D*). In contrast, bath application of SNAP-5,114 increased IPSC amplitude in response to optogenetic stimulation (134 ± 10% of control; *n* = 8 cells from 6 mice) and electrical stimulation (170 ± 7% of control; *p *= 0.008; Wilcoxon matched-pairs test), but to a lesser degree than for tiagabine [*F*_(1,38) _= 12.98; *p* = 0.0009; two-way ANOVA (drug); Fig. 3*C*]. A similar pattern was observed for charge transfer, with tiagabine displaying much stronger IPSC boosting than SNAP-5,114 [*F*_(1,39) _= 22.1; *p* < 0.0001 (drug); Fig. 3*D*]. Tiagabine prolonged the 20–80% rise time of GABA_B_R-mediated IPSCs, irrespective of whether from SSt INs or from the total population of INs (Fig. 3*E*), while SNAP-5,114 had no effect on onset kinetics (Fig. 3*F*). Likewise, tiagabine prolonged the decay time constants of all GABA_B_R-mediated IPSCs (Fig. 3*G*), while SNAP-5,114 did not (Fig. 3*H*). These data reveal that in DG circuits, heterosynaptic spillover of GABA is tightly regulated by neuronal reuptake (with a lesser role of astrocytes) to regulate GABA_B_R activation.

### Presynaptic GABA_B_Rs at EC inputs to DG are recruited by SSt INs to control inputs

GABA_B_Rs provide powerful presynaptic control to excitatory and inhibitory afferents by inhibiting Ca^2+^ influx (Kulik et al., 2018), which may be controlled by SSt IN activation in neocortex (Urban-Ciecko et al., 2015; Kanigowski et al., 2023). To determine whether SSt-dependent GABA release can control integration of synaptic information in DG, we next simultaneously stimulated perforant-path afferents with optogenetic stimulation to test the effect of SSt IN-mediated GABA_B_R inhibition on putative spatial inputs.

**Figure 4.**
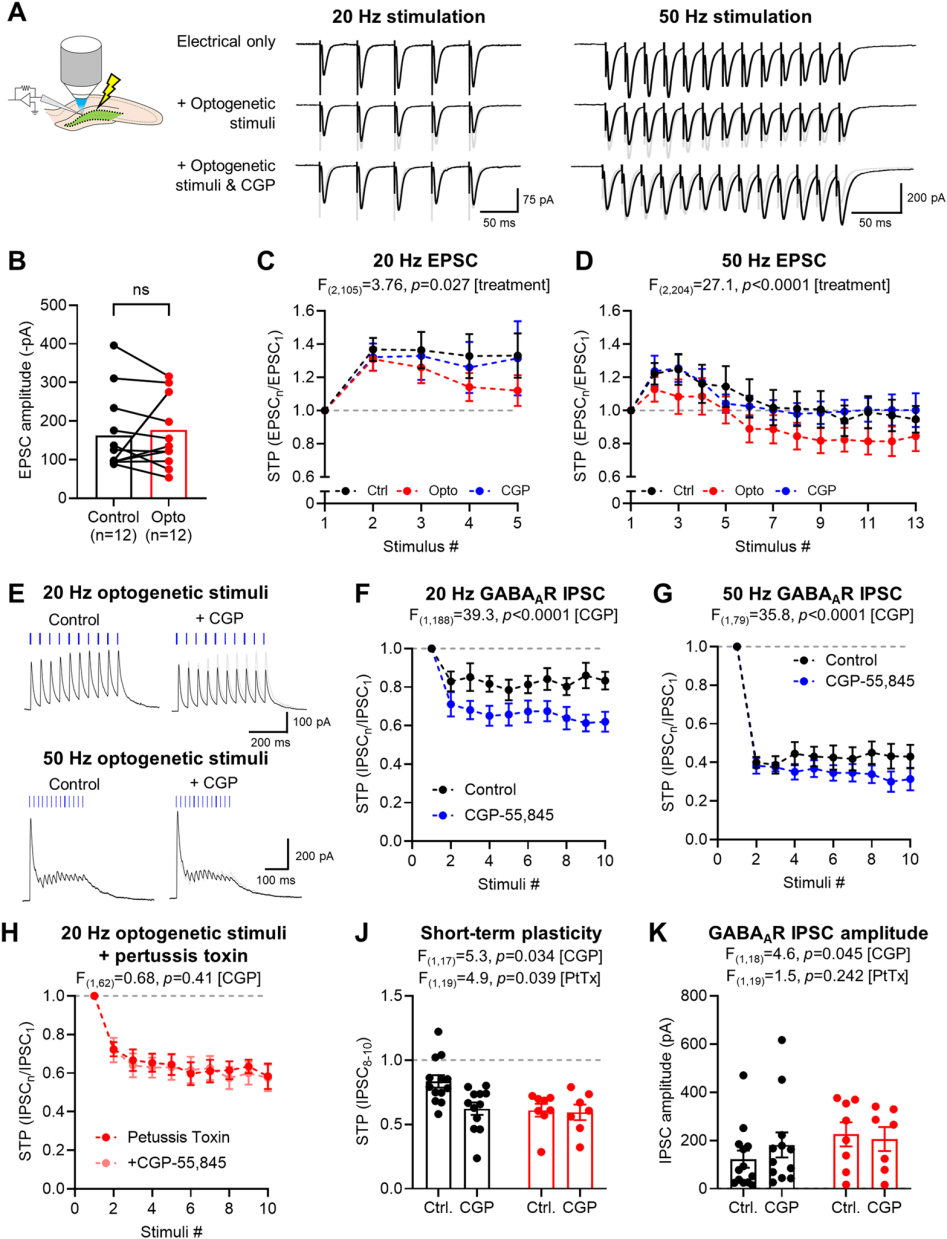
SSt INs recruit presynaptic GABA_B_Rs to control inhibit STP of spatial inputs to DG. ***A***, Schematic overview of experimental setup indicating combined optogenetic and electrical stimulation (left). Example traces from a recorded DGC in which GABA_A_Rs are blocked with 10 μM gabazine from a holding potential of −70 mV, using a Cs-gluconate–based internal solution, following either 20 Hz (5 stimuli) or 50 Hz (13 stimuli) electrical stimulation of the oML. Traces are shown from control conditions (black, top), combined with optogenetic stimuli (middle), or with optogenetic stimuli in the presence of CGP-55,845 (CGP, bottom). Control traces are shown for reference (gray). ***B***, Absolute EPSC amplitudes under control conditions (black) and combined with optogenetic stimulation (red) did not differ (*p* = 0.891; Wilcoxon matched-pairs test). ***C***, Quantification of STP of oML EPSCs following 20 Hz stimulation (*n* = 15 cells) in control (black), optogenetic stimulation (Opto; red), or optogenetic stimulation + CGP (CGP; blue), as compared with first EPSC amplitude (dashed gray line). ***D***, STP of oML EPSCs following 50 Hz stimulation (*n* = 11 cells), according to the same scheme as ***B***. ***E***, example GABA_A_R-mediated optogenetic stimulated IPSCs from SSt INs (blue bars) recorded in DGCs in response to 20 Hz (top) or 50 Hz (bottom), performed in the presence of 10 μM CNQX and 50 μM AP-5 from a holding potential of 0 mV with Cs-gluconate intracellular solution to block postsynaptic GABA_B_R-mediated K^+^ currents. Traces are shown for control recordings (left) or following bath application of 5 μM CGP-55,845 (CGP, right) compared with control recordings (gray traces). ***F***, STP of SSt-IN GABA_A_R-mediated IPSCs in response to 20 Hz optogenetic stimulation under control conditions (black) or following CGP application (blue) in 13 cells from 10 mice. ***G***, The same data, but plotted for 50 Hz optostimulation (*n* = 13 cells from 10 mice), with IPSCs further depressed after CGP application. ***H***, The same data as shown in ***F*** but following 2–3 h pretreatment with 500 μg/ml pertussis toxin (red) and following CGP application (pink, 8 cells from 4 mice). ***J***, Quantification of STP (IPSC_8–10_/IPSC_1_) under baseline conditions (black, 13 cells from 10 mice) or following 2–3 h pertussis toxin pretreatment (red, 8 cells from 4 mice). Data are shown from control (Ctrl.) or after CGP-55,845 application (CGP). ***K***, Modulation of optogenetic stimulated IPSC amplitudes by CGP as recorded under baseline conditions, or following pretreatment with pertussis toxin. All data is shown as mean ± SEM. Statistics are from two-way ANOVA, except panel ***B*** (Wilcoxon signed-rank test).

In the presence of gabazine to block potential effects of GABA_A_Rs, electrical stimulation was delivered at 20 Hz (5 stimuli) or 50 Hz (13 stimuli; Fig. 4*A*) to the oML while recording DGCs at −70 mV with a Cs^+^-based internal solution to block postsynaptic K^+^ currents. EPSCs evoked by perforant-path stimulation had amplitudes of 162.3 ± 29.0 pA. Amplitude of the first EPSC was not affected by either co-stimulation of oML and optogenetic activation of SSt INs (*p *= 0.520; Wilcoxon signed-rank test) or CGP application (*p *= 0.891; Wilcoxon signed-rank test; Fig. 4*B*), respectively, thus indicating tonic heterosynaptic GABA_B_R activation under baseline conditions. To induce STP of EPSCs, 20 Hz stimulation of perforant path inputs was performed in 15 cells from 11 mice. When concomitant optogenetic stimulation was given with oML stimulation, this facilitation was significantly reduced [*F*_(2,105) _= 3.760; *p *= 0.027; two-way ANOVA (drug); Fig. 4*C*]. This suppression of EPSC amplitude was blocked by bath application of CGP-55,845 (CGP; 5 μM), confirming that presynaptic GABA_B_Rs were responsible for this STP suppression. In contrast, 50 Hz perforant-path stimulation initially resulted in facilitating STP, which returned to baseline levels after five EPSCs (*n* = 11 cells from 9 mice; Fig. 4*D*). Concomitant SSt IN optogenetic stimulation largely prevented this facilitation and resulted in sustained depression of repetitive EPSCs, which was blocked by subsequent application of CGP [*F*_(2,351) _= 9.657; *p *< 0.0001; two-way ANOVA (treatment)].

As GABA_B_Rs can contribute to autoreceptor-mediated control of SSt IN presynaptic release (Booker et al., 2020), we performed a subset of recordings at 0 mV with the same optogenetic stimuli frequencies but without electrical stimulation (Fig. 4*E*). When recorded at 20 Hz, SSt IN-mediated GABA_A_R-mediated IPSCs were minimally depressing, which revealed greater short-term depression following CGP bath application [*F*_(1,188) _= 39.3; *p* < 0.0001; two-way ANOVA (treatment); Fig. 4*F*]. Under control conditions, 50 Hz stimulation of SSt INs produced greater short-term depression, which was also exacerbated by CGP bath application [*F*_(1,79) _= 35.8; *p* < 0.0001; two-way ANOVA (treatment); Fig. 4*G*]. Pretreatment of slices with pertussis toxin fully prevented any CGP-dependent change in STP [*F*_(1,62) _= 0.68; *p* = 0.41; two-way ANOVA (treatment); Fig. 4*H*]. Comparison of the STP (IPSC 8–10) and GABA_A_R-mediated IPSC amplitude revealed that pertussis toxin itself occludes GABA_B_R-dependent synaptic depression caused by CGP [*F*_(1,19) _= 4.9; *p *= 0.039; two-way ANOVA (pertussis toxin); Fig. 4*J*] and also occludes CGP-dependent enhancement of the first IPSC [*F*_(1,18) _= 4.6; *p* = 0.045; two-way ANOVA (CGP); Fig. 4*K*].

We found GABA_B_Rs activated by SSt INs contribute to the STP of perforant path inputs to the DG, as well as their own inhibitory strength. Our data indicate that SSt IN output is strongly regulated by tonic and activity-dependent autoreceptor activation, which require G_i/o_ protein activation. These data confirm that feedback SSt INs contribute to ongoing presynaptic inhibition of inputs to DG that likely contributes to gating of incoming spatial information.

## Discussion

We provide direct evidence that SSt INs are a major contributor to GABA_B_R inhibition of DG circuits, which is regulated by neuronal GABA uptake. Furthermore, we highlight the presence of sex differences in DGC excitability and GABA_B_R signaling. Finally, we provide direct evidence that presynaptic GABA_B_R activation by SSt INs controls the strength of incoming synaptic inputs from entorhinal cortex to the DG and by SSt INs themselves.

Hippocampal microcircuits are crucial for pattern separation of information, which requires sparse information transfer (Leutgeb et al., 2007; Schmidt-Hieber and Nolan, 2017). DG hilar INs (including SSt) are likely required for this process (Myers and Scharfman, 2009), but studies have largely focused on GABA_A_R signaling (Hefft and Jonas, 2005; Savanthrapadian et al., 2014; Guzman et al., 2021). DGCs, however, typically possess hyperpolarized membrane potentials that lead to fast and shunting inhibition (Bartos et al., 2001; Vida et al., 2006). GABA_B_Rs, in contrast, provide long-lasting, consistently hyperpolarizing and presynaptic inhibition to DGCs (Mott et al., 1993, 1999; Foster et al., 2013; Degro et al., 2015). Our data shows that feedback SSt INs contribute to GABA_B_R-mediated inhibition and may serve as an internal brake to regulate DGC activity, controlled by DGCs themselves. SSt IN activation of presynaptic GABA_B_Rs has been shown in active neocortical circuits (Urban-Ciecko et al., 2015; Kanigowski et al., 2023). Our data confirm that such recruitment of GABA_B_Rs by SSt INs is a likely common circuit motif across brain regions. In contrast, the DGCs display sparse firing rates and are capable of strongly recruiting SSt INs (Yuan et al., 2017). Our data provides evidence that SSt INs are also capable of recruiting high levels of postsynaptic GABA_B_R activation. Such inhibition within local circuits is postulated to contribute to the coherent timing of neuronal activity, particularly the nesting of theta, gamma, and ripple oscillations (Sik et al., 1997; Pangalos et al., 2013; Booker et al., 2020), which are sensitive to GABA_B_R activation (D'Antuono et al., 2005). How SSt IN-mediated GABA_B_R activation contributes to pattern separation and information coding in DG requires further investigation.

The source of GABA that drives GABA_B_R-mediated signaling likely originates from dendritic targeting INs (Scanziani, 2000; Booker et al., 2013; Gonzalez et al., 2018). Our data show that SSt INs contribute a significant portion of this GABA, in line with previous reports (Gonzalez et al., 2018). Indeed, our measured ∼60% viral transfection efficiency and whole-cell baclofen currents suggest that SSt INs activate up to 20% of GABA_B_Rs in DGCs. This complements the GABA originating from NGFCs in DG, which give rise to unitary GABA_B_R-mediated IPSCs (Armstrong et al., 2011) and are known to contribute more GABA_B_R signaling than SSt INs (Gonzalez et al., 2018). However, SSt INs are strongly recruited by DGC activity (Yuan et al., 2017) and have higher numbers in DG than neurochemically defined IN populations, including NGFCs (Jinno and Kosaka, 2006; Armstrong et al., 2011). As such, in vivo, SSt INs in the DG may lead to greater GABA_B_R activation to provide powerful feedback inhibition to DGCs on oML dendrites (Yuan et al., 2017). This result builds upon and extends previous findings suggesting a role for other INs, not just NGFCs, in producing GABA_B_R currents in PCs (Isaacson et al., 1993; Scanziani, 2000; Booker et al., 2013; Gonzalez et al., 2018). Our current findings also add further weight to the suggestion that multiple sources of GABA-mediated volume transmission exist. Importantly, such GABA sources likely rely on circuit-wide activation of many IN subtypes simultaneously or sequentially. Given the feedback nature of SSt INs, and their high connectivity with DGCs and hilar mossy cells (Savanthrapadian et al., 2014; Yuan et al., 2017), it is probable that they are recruited during behavior and their activation of GABA_B_Rs contributes to sparsification of spatial information (Myers and Scharfman, 2009; Hainmueller et al., 2024), plausibly explaining why pharmacological manipulation of GABA_B_Rs can limit spatial memory formation (Brucato et al., 1995) and regulate DG spiking (Foster et al., 2013). GABA_B_Rs likely play a critical role in controlling DGC activity, as at physiological membrane potentials they are consistently hyperpolarizing and exert strong presynaptic control; unlike GABA_A_Rs, which due to the relatively hyperpolarized membrane potentials of DGCs, largely exert shunting inhibition (Vida et al., 2006). Thus, GABA_B_R activation by SSt INs may contribute to circuit-level inhibition—particularly during exploration of novel environments (Hainmueller et al., 2024). We also show that GABA_B_R-mediated currents from SSt INs are regulated near-exclusively by neuronal GABA uptake and not astrocytes. The extent to which this is true for GABA_A_R IPSCs and whether DG SSt INs display astrocyte-dependent adenosine regulation as in CA1 (Matos et al., 2018; Shen et al., 2022) remains unknown. However, the role of astrocytes in DG GABA_B_R signaling is likely minimal given the failure of SNAP-5,114 to increase either amplitude or charge of SSt IN GABA_B_R-mediated IPSCs. This may highlight differences in circuit function of SSt INs (Hainmueller et al., 2024) and/or the role of astrocytes across hippocampal subfields (Matos et al., 2018).

Despite a lack of difference in the majority of measured electrophysiological parameters, we found heterogeneity in the decay kinetics of GABA_B_R-mediated IPSCs between male and female mice in IPSCs not originating from SSt INs. Neurogliaform INs contribute approximately twice the GABA_B_R-mediated currents to DGCs, compared with SSt INs (Gonzalez et al., 2018). Sex-specific differences in NGFCs have been reported in other brain structures (Gegenhuber et al., 2022), which may indicate that this is the source of altered GABA_B_R signaling. This adds to a growing body of literature highlighting sex-specific differences in GABA_B_R signaling at the neuronal (De Velasco et al., 2015) and behavioral levels (Campbell et al., 2002; Jeanblanc et al., 2023). The specific mechanisms underlying GABA_B_R kinetic modulation remain unclear; however, it is possible that altered expression of K-channel tetramerization domain proteins (KCTD) may be involved. In particular, KCTD12 confers longer GABA_B_R decay times (Fritzius et al., 2017; Booker et al., 2017b). Recent evidence has shown that KCTD12 contributes to aggressive behavior between female and male Drosophila (Williams et al., 2014). Whether such a mechanism exists in mammals is currently unknown.

In summary, we show that SSt INs significantly activate pre- and postsynaptic GABA_B_Rs in in the adult mouse DG, GABA released from SSt INs is regulated in the extracellular space by neuronal uptake mechanisms, and sex-specific differences in GABA_B_R kinetics exist. This highlights the importance of GABA_B_Rs in local circuit dynamics, particularly in the context of spatial information processing.

## Synthesis

Reviewing Editor: Lindsay De Biase, University of California Los Angeles Decisions are customarily a result of the Reviewing Editor and the peer reviewers coming together and discussing their recommendations until a consensus is reached. When revisions are invited, a fact-based synthesis statement explaining their decision and outlining what is needed to prepare a revision will be listed below. The following reviewer(s) agreed to reveal their identity: elise cope.

Inhibitory circuits are thought to contribute to the sparse activity in the hippocampal dentate gyrus. Inhibition arises from both fast, iontropic GABA signaling via GABA-A receptors as well as slow, metabotrophic GABA-B receptors. Yet, the contribution of specific inhibitory neuron populations to these GABA signaling mechanisms is unknown. Using optogenetics to silence hilar somatostatin+ inhibitory interneurons, they found that somatostatin+ inhibitory interneurons recruit GABA-B receptors to strongly inhibit dentate gyrus granule cells. This recruitment was found to be largely regulated by neuronal, but not astrocytic, GABA uptake mechanisms. They further showed that SST-mediated signaling can recruit presynaptic GABA-B receptors to regulate perforant path inputs onto granule cells. Taken together, their data suggests a mechanism by which somatostatin+ inhibitory interneurons contribute to the sparse activity of dentate gyrus granule cells. Additionally, the research reveals sex-specific differences in GABABR kinetics.

The authors present a clear hypothesis, the results are generally well described and the main conclusion that SSt INs contribute to dentate gyrus circuit function through GABAB receptors is convincing. While the work is interesting and fills a gap in knowledge, as prior work on SSTs focused mostly on GABAA responses. However, there are several concerns that need to be addressed.

Major comments:

1) Their statements that the INs that contribute to GABAB-mediated inhibition are poorly understood (i.e. the significance statement) are not correct. The role of PVs, SSt and nNos INs in targeting postsynaptic GABAB receptors in DG, along with involvement of Kir3 channels and GAT1 has previously been described (Gonzalez et al., J Neurosci 2018). Hence, there is existing literature showing that neurogliaform interneurons are the main source of GABAB-mediated signaling both in DG (Gonzalez et al) and in other brain regions (too many citations to mention). This is important to be more accurate about the current state of knowledge in the field in the introduction and discussion, especially in the section where they discuss the other interneuron sources. The authors should incorporate their findings into this existing literature more completely, especially as it provides more insight into the remaining GABABR activation generated by electrical stimulation that is discussed in the intro and discussion sections.

2) The authors compare fast and slow IPSCs peak and charge to conclude that "SSt INs directly recruit multiple GABA receptors, which provide divergent inhibition" (Line 218). While there is no doubt that these are inhibitory currents, here and throughout the manuscript, the terminology should be more precise since the authors have not tested how these synaptic currents generate inhibition (i.e. suppressing the excitability of downstream neurons).

3) Given that the journal's policy does not limit the number of figures, presenting all experiments as main figures would facilitate the reading of the article. For example,

regarding Figure 2 and supplemental figures 2-1 and 2-2, it is unclear why these figures were put into supplemental/extended data. It would be easier for the reader to combine these 3 figures into two main figures so that all the results are more accessible. This rearrangement would make possible to have a better comparison of the role of SST INs versus the total GABABR-mediated current. For example, Figure 2 panel F and Figure 2-1 panel D could be merged and the experiments with baclofen would take more relevance. Also, in Figure 2-1, panel E, it seems that the Y-axis should be "holding current" as I don't think it is a synaptic current.

4) There is some ambiguity in the presentation of the sex differences in the kinetics of GABABR synaptic currents. The authors "highlight the presence of sex differences in DGCs excitability and GABABR signaling" (line 335). However, the main figures do not show a significant difference, although they mention a specific difference in the action potential firing threshold of granule cells (Figure 1-1). First, it would be helpful if all data used to make central conclusions are included in the main figures. Second, it appears that there is no significant difference in the kinetics of currents activated by optogenetic activation of SSt INs, but only after electrical stimulation, presumably due to the activation of other subtypes of interneurons. This aspect should be addressed more clearly, and potentially the authors can make a stronger conclusion that rules out somatostatin interneurons in sex-dependent differences.

5) The authors show that GABA release from SSt INs exerts presynaptic control over STP of EPSCs and IPSCs via modulation by presynaptic GABAB receptors. Again, it would be easier to show all this data in main figures to avoid the need to refer to the supplemental material. In these experiments, currents are normalized to the peak of the first EPSC/IPSC, but it would be useful to also report the amplitude in CGP (i.e. Fig 3D) and assure that synaptic responses are stationary. The authors propose that postsynaptic GABAB signaling alters the amplitude of GABAA IPSCs, although I am somewhat dubious of the strength of this conclusion based on these small and variable effects with few n values. They could strengthen the conclusions about GABABR modulation of GABAA receptors by repeating it under conditions where GABAB-mediated G protein signaling is fully blocked to show that the regulation is absent.

Minor comments:

- Line 102 reports bilateral injections and line 154 reports contralateral hemisphere recordings, please clarify.

-Information about sources and titers of viruses used is missing from the methods.

-The methods indicate that only SSt primary was used - what is the purpose of the streptavidin?

-How many sections/cells were analyzed to determine the percentage of YFP+ SST cells?

-There is a typo in the results section - snap-5114 (not 5112) should be used to inhibit GAT3, etc.

-It would be helpful if all of the statistics were reported. In some cases only the significant ANOVA values are reported. Other main effects and posthoc values should be reported.

-Fig 1; It would be nice to show an overlay image of the YFP and SSt instead of the DAPI.

-Because pattern separation was not assessed, it should be removed from the results completely and only included in the discussion.

-It would be nice to provide some discussion on GABA-A signaling and how both GABA-A and GABA-B receptor signaling may work together to promote dentate gyrus sparse activity.

## Author Response

eN-NWR-0115-24: Response to reviewer's comments Synthesis Statement for Author (Required): Inhibitory circuits are thought to contribute to the sparse activity in the hippocampal dentate gyrus. Inhibition arises from both fast, iontropic GABA signaling via GABA-A receptors as well as slow, metabotrophic GABA-B receptors. Yet, the contribution of specific inhibitory neuron populations to these GABA signaling mechanisms is unknown. Using optogenetics to silence hilar somatostatin+ inhibitory interneurons, they found that somatostatin+ inhibitory interneurons recruit GABA-B receptors to strongly inhibit dentate gyrus granule cells. This recruitment was found to be largely regulated by neuronal, but not astrocytic, GABA uptake mechanisms. They further showed that SST-mediated signaling can recruit presynaptic GABA-B receptors to regulate perforant path inputs onto granule cells. Taken together, their data suggests a mechanism by which somatostatin+ inhibitory interneurons contribute to the sparse activity of dentate gyrus granule cells. Additionally, the research reveals sex-specific differences in GABABR kinetics. The authors present a clear hypothesis, the results are generally well described and the main conclusion that SSt INs contribute to dentate gyrus circuit function through GABAB receptors is convincing. While the work is interesting and fills a gap in knowledge, as prior work on SSTs focused mostly on GABAA responses. However, there are several concerns that need to be addressed. We thank the reviewers and editor for their positive appraisal of our work. In revising our manuscript, we have addressed the reviewers' comments both with changes to the text, figures, and inclusion of new data, which are outlined below. Major comments: 1) Their statements that the INs that contribute to GABAB-mediated inhibition are poorly understood (i.e. the significance statement) are not correct. The role of PVs, SSt and nNos INs in targeting postsynaptic GABAB receptors in DG, along with involvement of Kir3 channels and GAT1 has previously been described (Gonzalez et al., J Neurosci 2018). Hence, there is existing literature showing that neurogliaform interneurons are the main source of GABAB-mediated signaling both in DG (Gonzalez et al) and in other brain regions (too many citations to mention). This is important to be more accurate about the current state of knowledge in the field in the introduction and discussion, especially in the section where they discuss the other interneuron sources. The authors should incorporate their findings into this existing literature more completely, especially as it provides more insight into the remaining GABABR activation generated by electrical stimulation that is discussed in the intro and discussion sections. The reviewer makes an important point. Indeed, the role of neurogliaform cells should not be underestimated. As shown in Gonzalez et al., 2018, the contribution from these cells is indeed large. We have now included additional text to the introduction (lines 80-83) and discussion (lines 368-382) to address this. 2) The authors compare fast and slow IPSCs peak and charge to conclude that "SSt INs directly recruit multiple GABA receptors, which provide divergent inhibition" (Line 218). While there is no doubt that these are inhibitory currents, here and throughout the manuscript, the terminology should be more precise since the authors have not tested how these synaptic currents generate inhibition (i.e. suppressing the excitability of downstream neurons). We have now referred only to GABAAR and GABABR mediated IPSCs throughout the figures and results to avoid confusion. In the revised discussion, we now reflect on how such currents may lead to altered circuit function. 3) Given that the journal's policy does not limit the number of figures, presenting all experiments as main figures would facilitate the reading of the article. For example, regarding Figure 2 and supplemental figures 2-1 and 2-2, it is unclear why these figures were put into supplemental/extended data. It would be easier for the reader to combine these 3 figures into two main figures so that all the results are more accessible. This rearrangement would make possible to have a better comparison of the role of SST INs versus the total GABABR-mediated current. For example, Figure 2 panel F and Figure 2-1 panel D could be merged and the experiments with baclofen would take more relevance. Also, in Figure 2-1, panel E, it seems that the Y-axis should be "holding current" as I don't think it is a synaptic current. We have now re-organised the figures incorporating Extended data 2-1 into Figure 2 and Extended data 2-2 into a new Figure 3 which directly compares the effects of astrocytic and neuronal GABA uptake mechanisms on SSt IN mediated GABABR IPSCs. Likewise, we have now incorporated Extended data 3-1 into a combined new Figure 4, assessing the role of presynaptic GABABRs on DG function. We have corrected the error in former Panel 2-1E. 4) There is some ambiguity in the presentation of the sex differences in the kinetics of GABABR synaptic currents. The authors "highlight the presence of sex differences in DGCs excitability and GABABR signaling" (line 335). However, the main figures do not show a significant difference, although they mention a specific difference in the action potential firing threshold of granule cells (Figure 1-1). First, it would be helpful if all data used to make central conclusions are included in the main figures. Second, it appears that there is no significant difference in the kinetics of currents activated by optogenetic activation of SSt INs, but only after electrical stimulation, presumably due to the activation of other subtypes of interneurons. This aspect should be addressed more clearly, and potentially the authors can make a stronger conclusion that rules out somatostatin interneurons in sex-dependent differences. We have now included electrical stimulation data in the main figures of the manuscript, which highlights the sex-specific differences in GABABR kinetics at the circuit level. We have included further discussion of these differences (lines XX, YY). 5) The authors show that GABA release from SSt INs exerts presynaptic control over STP of EPSCs and IPSCs via modulation by presynaptic GABAB receptors. Again, it would be easier to show all this data in main figures to avoid the need to refer to the supplemental material. In these experiments, currents are normalized to the peak of the first EPSC/IPSC, but it would be useful to also report the amplitude in CGP (i.e. Fig 3D) and assure that synaptic responses are stationary. The authors propose that postsynaptic GABAB signaling alters the amplitude of GABAA IPSCs, although I am somewhat dubious of the strength of this conclusion based on these small and variable effects with few n values. They could strengthen the conclusions about GABABR modulation of GABAA receptors by repeating it under conditions where GABAB-mediated G protein signaling is fully blocked to show that the regulation is absent. In addressing the reviewer's suggestion, we have incorporated the supplementary materials into a new Figure 4. We agree with the reviewer that these original data were somewhat underpowered. To this end, we have performed additional experiments to increase the statistical power of these data, which strengthen our original data (Figure 4F &4G). Additionally, in parallel with these new experiments, we performed pertussis toxin pre treatment of a subset of SSt-ChR2 brain slices. In these slices we observe no CGP dependent alteration of SSt IN GABAAR IPSCs (Figure 4H), these data combined with new analyses displaying the 1st IPSC amplitude changes following CGP application (Figure 4K), indicate that STP effects are more likely due to a combination of tonic inhibition and reduced release probability, due to GABABR activation. The results have been reworded to reflect this. Minor comments: - Line 102 reports bilateral injections and line 154 reports contralateral hemisphere recordings, please clarify. We performed both bilateral and unilateral injections. When unilateral injections were performed, recordings were made in the contralateral hemisphere that expressed few (if any) obvious SSt IN axons. -Information about sources and titers of viruses used is missing from the methods. This has now been included. -The methods indicate that only SSt primary was used - what is the purpose of the streptavidin? This was to visualise recorded neurons. A comment has been added to the methods (line 213-214) -How many sections/cells were analyzed to determine the percentage of YFP+ SST cells? 1 slice per mouse was used for quantifying ChR2 expression. This has now been stated in the methods (Line 219). -There is a typo in the results section - snap-5114 (not 5112) should be used to inhibit GAT3, etc. These typographical errors have now been corrected. -It would be helpful if all of the statistics were reported. In some cases only the significant ANOVA values are reported. Other main effects and posthoc values should be reported. We have now included on all graphs the outcome of main effects from ANOVA tests. The results of all pair-wise statistical tests are reported in figure legends. -Fig 1; It would be nice to show an overlay image of the YFP and SSt instead of the DAPI. We have now replaced Figure 1A with an overlay of ChR2-YFP and SSt. We have retained the DAPI stain in the higher magnification insets for reference. -Because pattern separation was not assessed, it should be removed from the results completely and only included in the discussion. Any reference to pattern separation in the results has been removed. -It would be nice to provide some discussion on GABA-A signaling and how both GABA-A and GABA-B receptor signaling may work together to promote dentate gyrus sparse activity
